# The direct and indirect costs of both overweight and obesity: a systematic review

**DOI:** 10.1186/1756-0500-7-242

**Published:** 2014-04-16

**Authors:** Anne Dee, Karen Kearns, Ciaran O’Neill, Linda Sharp, Anthony Staines, Victoria O’Dwyer, Sarah Fitzgerald, Ivan J Perry

**Affiliations:** 1 Department of Public Health, Health Service Executive West, Mount Kennett House, Henry Street, Limerick, Ireland; 2 Department of Epidemiology and Public Health, Western Gate Building, University College Cork, Cork, Ireland; 3 J.E. Cairns School of Business and Economics, National University of Ireland, Galway, Ireland; 4 National Cancer Registry Ireland, Building 6800, Cork Airport Business Park, Kinsale Road, Cork, Ireland; 5 School of Nursing and Human Sciences, Dublin City University, Dublin 9, Ireland

**Keywords:** Direct costs, Health care costs, Indirect costs, Lost productivity costs, Overweight, Obesity

## Abstract

**Background:**

The rising prevalence of overweight and obesity places a financial burden on health services and on the wider economy. Health service and societal costs of overweight and obesity are typically estimated by top-down approaches which derive population attributable fractions for a range of conditions associated with increased body fat or bottom-up methods based on analyses of cross-sectional or longitudinal datasets. The evidence base of cost of obesity studies is continually expanding, however, the scope of these studies varies widely and a lack of standardised methods limits comparisons nationally and internationally. The objective of this review is to contribute to this knowledge pool by examining direct costs and indirect (lost productivity) costs of both overweight and obesity to provide comparable estimates. This review was undertaken as part of the introductory work for the Irish cost of overweight and obesity study and examines inconsistencies in the methodologies of cost of overweight and obesity studies. Studies which evaluated the direct costs and indirect costs of both overweight and obesity were included.

**Methods:**

A computerised search of English language studies addressing direct and indirect costs of overweight and obesity in adults between 2001 and 2011 was conducted. Reference lists of reports, articles and earlier reviews were scanned to identify additional studies.

**Results:**

Five published articles were deemed eligible for inclusion. Despite the limited scope of this review there was considerable heterogeneity in methodological approaches and findings. In the four studies which presented separate estimates for direct and indirect costs of overweight and obesity, the indirect costs were higher, accounting for between 54% and 59% of the estimated total costs.

**Conclusion:**

A gradient exists between increasing BMI and direct healthcare costs and indirect costs due to reduced productivity and early premature mortality. Determining precise estimates for the increases is mired by the large presence of heterogeneity among the available cost estimation literature. To improve the availability of quality evidence an international consensus on standardised methods for cost of obesity studies is warranted. Analyses of nationally representative cross-sectional datasets augmented by data from primary care are likely to provide the best data for international comparisons.

## Background

The prevalence of overweight (BMI > 25 Kg/m^2^) and obesity (BMI > 30 Kg/m^2^) is rising internationally [1]. As BMI increases, so too does the prevalence of co-morbid conditions including diabetes, cardiovascular disease (CVD) and some cancers [2]. Rising medical costs are a matter for concern globally, and many attempts have been made to estimate the costs associated with the increasing prevalence of both overweight and obesity [3-8]. This review examines the literature that has focussed on both the direct and indirect costs of overweight and obesity.

The healthcare costs associated with obesity are typically calculated using two main approaches, a top down method based on estimation of population attributable fractions (PAF method) and two “bottom up” methods, based on analyses of cross-sectional and longitudinal datasets respectively. Other methods such as simulation modelling can also be used to estimate costs of overweight and obesity, however this review will focus on the PAF method and analyses of cross-sectional and longitudinal datasets. The PAF method is based on the use of nationally available prevalence data for obesity and/or overweight, to which relative risk estimates for the relevant co-morbid conditions are applied, thereby producing estimates of the population attributable fraction (PAF) for each condition. The PAFs are applied to national cost data for the relevant conditions to give an overall estimate of the cost of overweight and obesity. Bottom up approaches draw on existing individual level data from cross-sectional datasets or from longitudinal studies to link BMI measurements with data on health care utilisation patterns and or sickness related absenteeism [9-12]. The issue of presenteeism is also important to consider when calculating the cost of overweight and obesity. Presenteeism refers to a situation in which the employee remains in the workforce but their productivity is adversely affected by their health condition – for example as a result of their mobility being reduced. The additional service utilisation associated with overweight and obesity is determined by multivariate regression analysis and monetised using cost data for the country concerned. The longitudinal approach may provide the most accurate estimates of the cost of overweight and obesity as the occurrence of health outcomes and sickness related absenteeism is measured directly in a group of individuals who are followed over time. However, such data are rare and resource intense in collection, the duration of follow-up required to accrue sufficient events is typically in decades and participants in longitudinal cohort studies are often poorly representative of the wider population, thereby constraining the generalisability of the findings.

There is now an extensive literature on the cost of obesity. Many of the early cost of obesity studies used a prevalence based top down PAF approach. However bottom up approaches have been used more widely in recent years. There are numerous factors which have resulted in considerable heterogeneity existing within the literature on cost of obesity studies, including:

•The scope of studies varies with some estimating the cost of obesity alone and some including the cost of overweight. Variation is also evident with respect to the number of conditions included, for example, which ranged from four [9,10], to ten or more [11,12].

•Direct healthcare costs are most commonly calculated, although some studies also include indirect costs, mainly based on productivity losses due to illness and disability and early mortality. Even within the direct healthcare cost there is variation between studies, with some addressing a wide range of therapies and others narrowly focussed on a single aspect, e.g. drug costs [13] or hospital costs [14,15].

•Different approaches are taken to the presentation of estimated costs. These include absolute figures in the currency of the country, the proportion of the national expenditure on healthcare that can be attributed to overweight/obesity or relative per capita spend by BMI category. In the early 1990s, the World Health Organisation (WHO) categories for BMI of underweight, normal weight, overweight and obese were not agreed or consistently applied internationally. Thus in the earlier studies there is also variation in the BMI categories used to describe overweight and obesity.

•The perspective of the studies differ as each country has a different healthcare system, with various “bundles of basic care”, range of services and the balance between public and private sector funding for health care.

These factors hinder international comparisons in cost of overweight and obesity studies. In 1997 however, the WHO standardised the definition of normal weight, overweight and obesity [16], and most studies after 1997 have adopted these definitions.

Many studies of the cost of obesity have been carried out in the US. This is unsurprising given the scale of the problem (over 60% of the US population are either overweight or obese [17]) and the availability of detailed databases to support research on the subject. The latter include the National Health and Nutrition Examination Survey (NHANES) which was established in the 1970s and provides data on BMI prevalence and trends over time. Other databases include the Medical Expenditure Panel Survey (MEPS) which provide data on healthcare usage and expenditures on the non-institutionalised American civilian population. Estimates from the 1990 suggested that in the US between 4.3% [18] to 7.8% [19] of total healthcare expenditure could be attributed to obesity. Early studies from other jurisdictions include Australia [20], New Zealand [21], Canada [11], France [12,22], The Netherlands [23] and the UK [24]. Direct healthcare costs were the main focus of many of these studies [11,20-24], mainly using the top down approach [11,12,20,21,23] and most focussed only on obesity [11,12,20-22,24]. Of those studies presenting their results as a percentage of healthcare expenditure, the range was from 1% [24] to 4% [23], being generally lower than the values calculated in the US studies. Due to the inconsistencies highlighted above, cross country comparisons are difficult. However it would appear that the proportion of healthcare costs attributable to overweight and/or obesity in the US is somewhat higher than in other developed countries.

While a number of reviews have been published [3-8,25-30] it is important in the context of a rapidly emerging evidence base that these are updated on a regular basis. These previously published reviews have attempted to assimilate large amounts of data from heterogeneous studies which greatly limits the extent to which conclusions or recommendations can be drawn from them. This review attempted to examine a more homogenous group of studies which investigated both the direct and indirect costs of overweight and obesity and if comparability of such studies can be improved by reducing the scope of the search strategy. Therefore this review explores the problems associated with the methodological heterogeneity of these types of studies, even within the narrow scope of this review. There is a substantial lack of literature examining the full scope of costs associated with overweight and obesity. As a means to address literature gap and add to the current body of evidence, this review attempts to reveal the most appropriate methodologies for these studies and the most appropriate data sources to be used which would enable international comparisons.

This review formed part of the preliminary work for the Irish cost of overweight and obesity study which estimated the direct and indirect costs for overweight and obesity in Ireland for 2009. The study began in 2011 and was completed in September 2012. Preliminary work for this study included a broader look at literature estimating the cost of overweight and obesity. Literature which was published after 2001 and up to the end of 2011 was examined and this timeframe is reflected in the searching and identification of relevant papers for the current review [31]. Articles published prior to 2001 were not eligible for inclusion as economic data which is older than ten years bares little relevance in the changing economic climate.

## Methods

### Data sources and search strategy

This review has been guided by the PRISMA (Preferred Reporting Items for Systematic Reviews and Meta-Analyses) statement. An extensive search of the literature was conducted using the PubMed, EMBASE, CINAHL, Science Direct, and ProQuest databases to identify relevant studies. Various combinations of the terms ‘cost’, ‘overweight’ and ‘obesity’ were used, and these terms were expanded for a full MESH search of PubMed. In order to provide a thorough breakdown of articles according to scope, two main searches were conducted in PubMed. The first search focused on the direct cost of overweight or obesity, with the second search focusing on the indirect cost of overweight and obesity.

Search 1: Included a MeSH search of the following search terms: (Overweight OR Obesity OR Obesity, Abdominal OR Anti-Obesity Agents OR Obesity, Morbid OR Abdominal obesity metabolic syndrome OR Anti-Obesity Agents) AND (Costs and Cost Analysis OR Economics OR economics OR Cost-Benefit Analysis OR Cost Allocation OR Cost of Illness OR Cost Control OR Health Care Costs OR Direct Service Costs OR Hospital Costs OR Employer Health Costs OR Drug Costs).

Search 2: In PubMed a MeSH search of the following search terms: (Overweight OR Obesity) AND (Cost OR Absenteeism OR Presenteeism OR Productivity OR Sick Leave OR Illness Benefit OR Cost to Employer OR Workers’ Compensation OR Disability OR Premature Mortality).

The search in CINAHL used the terms ‘obesity and cost’ or ‘overweight and cost’ and the search in EMBASE was searched using the terms ‘overweight’ or ‘obesity’ and ‘cost’. Science Direct was searched using the terms ‘overweight’ or ‘obesity’ and ‘cost*’ included in the title, abstract or as a keyword in the article. The search in ProQuest used the terms ‘overweight and cost*’ or ‘obesity and cost*’. Reference lists of retrieved articles were fully scanned and a thorough search of the grey literature was conducted to include national reports relating to the cost of overweight and obesity.

Furthermore Conference Proceedings was searched for unpublished abstracts. Government websites for the major developed countries were searched to identify relevant reports; this was augmented with a Google search. The Cochrane Library was also searched for cost of obesity studies. Reference lists of retrieved reports and articles were scanned to identify any further potential studies that had been missed. Review articles were identified and while they did not form part of the review, their reference lists were searched for further unidentified articles.

Limits applied to the search strategy include; studies published from 2001 to end of 2011; English language; Human; Publication type included Conference, Doctoral Dissertation, Journal Article, Masters Thesis, Meta-Analysis, Proceedings, Review and Systematic Review. No restrictions were placed on the study population nationality, or statistical designs or methods.

#### Eligibility criteria

Studies generated from the search strategy were deemed eligible for inclusion in the review if they satisfied all of the strict exclusion criteria which included:

1. Studies focused on children and other discreet groups (e.g., women only, truck drivers etc.). Children were excluded as they are not included in the Irish cost of overweight and obesity study for which this literature review was performed.

2. Studies published before 2001 or after 2011.

3. Studies reporting small study populations (cohort size <500 overweight or obese persons).

4. All commentary and review articles.

5. Studies which did not measure BMI by the WHO standard measures as this would make comparisons very difficult.

The results were summarised and presented in each case as the costs determined in the study, and these costs were also converted to 2009 Irish Euros using inflation rates and Purchasing Power Parity (PPP) in order to improve comparability.

## Results

### Search strategy results

From PubMed 3481 articles were retrieved from the combined searches. The combined additional searches of EMBASE, CINAHL, Science Direct, and ProQuest yielded 4249 articles. Following removal of duplicates there were 2283 articles to be reviewed. Following review by title, 2042 articles were deemed ineligible. The abstracts of the remaining 241 articles were then reviewed and assessed for inclusion. This resulted in 194 articles being excluded and the full text of 45 reports and articles on the cost of overweight and/or obesity were accessed and read in detail to determine eligibility. Following full text review, 38 studies were excluded according to the specified eligibility criteria.

**Figure 1:**
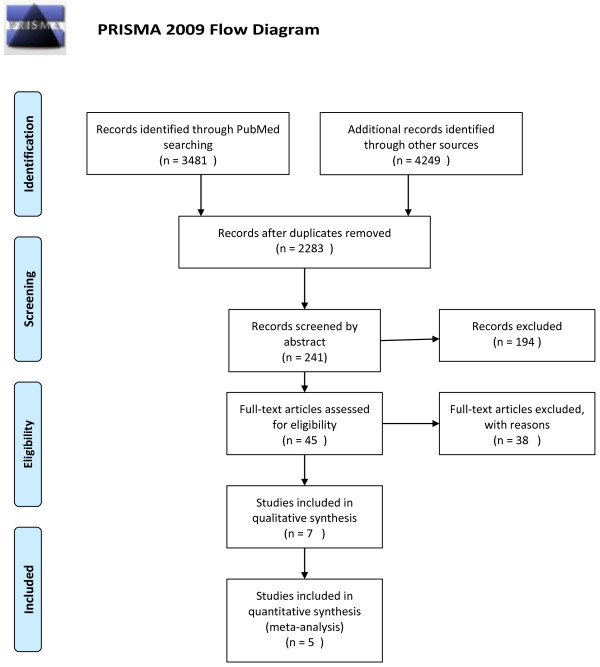
PRISMA 2009 flow diagram.

The breakdown of articles by scope was direct costs of obesity (6 studies), direct costs of overweight and obesity (22 studies), direct and indirect costs of obesity (7 studies), direct and indirect costs of overweight and obesity (7 studies) and indirect costs only (3 studies). Only those studies that addressed the direct and indirect costs of both overweight and obesity were included in this review. In total, 7 studies attempted to estimate the direct and indirect costs of overweight and obesity [32-38]. Of these, two did not measure indirect costs, looking instead at transfer payments [34], and out of pocket expenses [38]. These were excluded, leaving only 5 articles published since 2001 estimating the direct and indirect costs of overweight and obesity. This search strategy is outlined in Figure 1.

### Measuring direct and indirect costs

Three studies used the top down approach of calculating PAFs for both direct and indirect costs [32,36,37]. Two of these studies [32,36] applied the human capital approach to calculating the indirect costs. This approach estimates the value of lost production as a result of work absences from the employees’ perspective. Output lost is valued using the individual’s work related income and the full duration of their absence from the workplace. The third study, that of Schmid and colleagues provides little detail on how indirect costs were calculated [37].Finklestein et al. in the US used a cross-sectional approach to both direct and indirect costs [35] whereas Borg et al. [33] used data from a longitudinal cohort,

Of the studies that used the PAF methodology [32,36,37] all three included a wide range of co-morbid illnesses, but the calculated attributable fractions differed for each study. Anis et al. [32] calculated PAFs for both overweight and obesity and for males and females separately. Konnopka et al. [36] calculated PAFs for males and females separately, but combine overweight and obesity, while Schmid et al. [37] calculate PAFs separately for overweight and obesity but combine males and females. All three studies included a wide range of activities in the direct cost calculation, including inpatient, outpatient and pharmaceutical costs, details of which are outlined in Table 1.

**Table 1:** Direct and indirect costs of overweight and obesity

**Study**	**Costs included direct**	**Cost included indirect**	**Type of study**	**Results direct**	**Results indirect**	**Results total (€ 2009)**	**Percentage of results that are indirect**
**Anis et al. 2010 **[32]**, Canada**	Hospital inpatient and outpatient visits, physician services, drug costs, health research and other health care	Morbidity due to both long and short-term disability	Prevalence based PAF	CA$5.96 billion	CA$5 billion	$10.96 billion (€7.3 billion 2009)	54%
**Konnopka et al. 2011 **[36]**, Germany**	Inpatient and outpatient treatment, rehabilitation and non-medical costs (administration, research etc)	Sickness absence, early retirement and mortality using human capital approach	Prevalence based PAF	€4.854 billion (2.1% of total healthcare costs for 2002)	€5.019 billion	€9.873 billion (€11.01 billion 2009)	51%
**Schmid et al. 2004 **[37]**, Switzerland**	All healthcare costs for obesity and co-morbid conditions	Work absenteeism, early retirement and premature death relating to co-morbidities	Prevalence based PAF	N/A	N/A	CHF2.69 billion (€1.91 billion 2009)	N/A
**Finkelstein et al. 2010 **[35]**, US**	All Medical costs	absenteeism and presenteeism	Cross-sectional	$30.3 billion	$42.8 billion	$73.1 billion (€51.92 billion 2009)	59%
**Borg et al. 2005 **[33]**, Sweden**	Hospital inpatient costs only	Lost productivity due to increased mortality	Longitudinal cohort	SEK Billion: 2.17	SEK Billion: 2.93	SEK billion: 5.1 (€0.54 billion 2009)	58%

There is further variation in the calculation of indirect costs. Two of the studies [32,36] applied the human capital approach to calculating the indirect costs. Output lost is valued using the individual’s work related income and the full duration of their absence from the workplace. The third study, that of Schmid and colleagues provides little detail on how indirect costs were calculated [37]. While Anis et al. [32] measure long and short term disability, Konnopka et al. [36] also measure early retirement and premature mortality. The latter also include paid and unpaid work in their calculation of indirect costs. Schmid et al. [37] measure sickness absence, early retirement and premature mortality productivity losses, but the methodology used in this part of the study is unclear.

While the results for the Canadian study [32] and the German study [35] are remarkably similar in terms of absolute costs, Table 1, it must be remembered that the population of Germany is over twice that of Canada (82 million vs. 33 million). Thus it would appear that the Canadian estimates of both direct and indirect costs are disproportionately high. However this most likely reflects differences in the two healthcare systems and methodological differences between the two studies. The Canadian estimates were based on BMI prevalences derived from measured height and weight whereas the source of the BMI data in the German study is unclear. The findings from Switzerland (population 7 million) are broadly proportionate with those from Germany.

For Sweden, Borg et al. [33] estimated direct costs based on hospital inpatient costs only and indirect costs were estimated on the basis of lost productivity due to increased mortality. They studied a longitudinal cohort of adult men and women from the Malmö Prevention Project, enrolled between 1974 and 1984 and followed up for 15 years. BMI was based on measured height and weight in study subjects. Using a stepped regression modelling technique, they estimated costs for the normal weight, overweight and obese groups. They found no major differences in hospital usage between the overweight and normal weight groups and consequently there were no significant costs differences between these groups. By contrast there were significant cost differences between normal weight groups and obese groups. Similarly, when looking at the lost productivity and associated costs, the main differences lay between the obese group and the others and there was little difference between the overweight and normal weight groups. Notably, the study did not include drug or other healthcare costs in the estimate of costs. The low costs estimated in this study reflect the size of the Swedish population (9 million) and the limited scope of the study which was confined to hospital costs and premature mortality. However these estimates derived from a longitudinal cohort study are arguably closer to reality than those derived from top down estimations of population attributable fractions.

In the US in 2010, Finklestein et al. [35] used data from MEPS (for healthcare costs) and the National Health and Wellness Survey (NHWS) (for data on absenteeism and presenteeism) to estimate costs on a cross sectional basis. BMI was based on self-reported data. For direct costs they measured all healthcare use, and for indirect costs they measured absenteeism and presenteeism as determined in self-reported responses in the NHWS. They found that for all categories of obesity, the three variables: healthcare costs, absenteeism and presenteeism all increased with increasing BMI. These findings did not apply to overweight men, who had lower rates of presenteeism than their normal weight counterparts. They also found that although people with a BMI > 35 kg/m^2^ represent only 37% of all obese people in the US, they account for 61% of the costs. Presenteeism was found to be a stronger driver of costs associated with lost productivity than was absenteeism. The US cost estimates are high, reflecting their population size , but they are consistent with those from Germany [36].

Table 1 provides a breakdown of the five studies. Indirect costs were higher than direct costs in all four of the five studies which presented separate estimates for direct and indirect costs.

## Discussion

Healthcare costs increase as BMI increases, and so do costs associated with lost productivity. The costs associated with lost production are higher than direct healthcare costs. There is a substantial international literature on the question of the costs associated with weight gain but review and synthesis of this literature are hindered by the heterogeneity of the studies in terms of scope, data sources, data quality and methodological approaches. This was evident even within the small number of studies that met the criteria for inclusion in this review.

The available evidence suggests that increasing BMI is associated with increased healthcare consumption and reduced productivity. While the risk of co-morbidity is greatest in the obese segment of the population (approximately 25% of adults in many developed countries) there is a graded relation between BMI and major causes of co-morbidity. Therefore it may be deduced that the additional larger segment of the population who are overweight (approximately 40% of adults in developed countries) would be the main driver of costs. Based on current evidence, this is not the case. It is also clear that the per capita costs increase as BMI increases, with obesity accounting for much higher costs than overweight. In Borg et al’s longitudinal study in Sweden, obesity was the main driver of costs [33]. Similarly, Finklestein et al. found that healthcare costs, absenteeism and presenteeism were all increased in the obese whereas costs in overweight men were not increased relative to those of normal weight [35].

Estimation of the costs of illness based on population attributable fractions poses particular difficulties. The outputs from this exercise depend critically on four core inputs which vary considerably in the precision of estimates and the inclusion criteria applied in different studies: the estimated prevalence of overweight and obesity, the list of co-morbid conditions linked to overweight and obesity, the relative risk estimates used to calculate PAF’s and the availability and quality of national cost data. There are additional concerns in relation to the PAF based approach, including the problem of double counting due to multi-morbidity. Double counting is an issue associated with the second of the core inputs outlined previously. It is imperative that diagnoses of the same health issue (co-morbid condition) associated with overweight and obesity are grouped into one episode of care to avoid double counting. While these studies may have some merit in highlighting for policy makers the relative scale of specific problems and the need for investment in prevention, the heterogeneity evident in this narrowly focused literature review raises important questions on the validity and reliability of this approach.

The analysis of cross-sectional datasets offers a more promising avenue of investigation. Most developed countries now carry out regular national health and lifestyle surveys to monitor the health and wellbeing of the population and assess the impact of public health policies and interventions [39,40]. These cross-sectional studies involving relatively large and representative samples of adults provide valuable opportunities to measure BMI and other measures of body fat with linked data on health care utilisation in primary and secondary care, absenteeism and presenteeism during the preceding year. The development and standardisation of minimum datasets for cost of illness studies drawing on national health and wellbeing surveys would represent a significant advance in this area. Ideally, analyses of cross sectional data from representative population samples should be supplemented with high quality routine primary care data and data from longitudinal studies on incident disease and mortality. Theoretically the use of longitudinal data with measured BMI and data on morbidity, health care utilisation, mortality and productivity loss offer the best opportunity for obtaining good quality data on the costs of overweight and obesity. In practice however for most countries worldwide, the relevance, quality and timeliness of cost of illness data from longitudinal studies is limited.

A number of conclusions may be drawn on the estimation of indirect costs. Presenteeism is rarely measured. It was addressed in only one of the studies [35] included in this review and in few studies in the wider literature [41,42]. As the concept of presenteeism is inherently subjective the findings in relation to this issue may not be reliable or generalisable. There is clearly a need for ongoing work on the development and validation of appropriate instruments in this area. By contrast, absenteeism is easier to measure and more likely to be verifiable from alternative datasets (e.g. social welfare data, hospital in-patient days), making it a more useful marker of lost productivity. Premature mortality was measured in only three of the studies [33,36,37], all of which used different methods. Despite the methodological weakness and inconsistencies of these studies it noteworthy however in the four studies which presented separate estimates for direct and indirect costs, indirect costs were higher, accounting for between 54% and 59% of the estimated total costs.

A limitation of this review is the narrow eligibility criteria for inclusion of studies. As the objective of this study was to review studies which focused on both direct and indirect costs only, the scope of the search strategy was significantly narrower. However, the dearth of literature examining the full scope of costs associated with overweight and obesity is an additional factor to this limitation. Further to this paucity of literature, the absence of international standardised methods and a lack of consensus in the design of these cost of overweight and obesity studies, hinders the completion of a comprehensive review.

## Conclusion

While cost of overweight and obesity studies are helpful for health program planning in terms of quantifying the magnitude of the problem and setting funding priorities it is clear that the results of these studies merit cautious interpretation, particularly with regards how best to tackle the problem. Furthermore these studies need to be considered within the broader context of work on the relative cost-effectiveness of potential policies, programmes and interventions addressing the ongoing epidemic of overweight and obesity in children and adults. In conclusion, there is a consensus that as BMI rises, so too do both the direct healthcare costs and the indirect costs due to reduced productivity and early mortality. A more precise quantification of the increase in costs is hindered by the heterogeneity in the evidence-base even when studies with a similar scope are considered. To improve the quality of information available for policy and planning purposes, greater standardisation is required both in methodological approach and reporting. Moreover, while top-down studies are of value in the absences of other data, it is likely that investment in high-quality, bottom-up cross-sectional and longitudinal studies will be needed to better understand the main drivers of costs and target prevention strategies and/or interventions most appropriately.

### Key points

•Heterogeneity is a major limitation in the cost of overweight and obesity literature.

•Bottom up approaches to cost of obesity studies help to identify the main drivers of cost and therefore can inform focussed public health interventions.

•While cost of overweight and obesity studies are useful to highlight the scale of the problem, they need to be set within the wider context of work on the relative cost-effectiveness of potential policies, programmes and interventions.

## Competing interests

The authors declare that they have no competing interests.

## Authors’ contributions

AD carried out the review and wrote the manuscript. KK assisted in the search strategy, review process and reviewed the manuscript. CON reviewed the manuscript and had a supervisory role in the review process. LS reviewed the manuscript and contributed to the review process. AS reviewed the manuscript. VOD assisted in the review process and reviewed the paper. SF assisted in writing, revising and reviewing the manuscript. IJP was the Principal Investigator on the overall study, advised in the review process, assisted in drafting and reviewing manuscript. All authors read and approved the final manuscript.
